# Current Trends on Role of Biological Treatment in Integrated Treatment Technologies of Textile Wastewater

**DOI:** 10.3389/fmicb.2021.651025

**Published:** 2021-03-25

**Authors:** Maria Belen Ceretta, Débora Nercessian, Erika A. Wolski

**Affiliations:** ^1^Biochemical Engineering Group, Institute of Science and Technology of Food and Environment (INCITAA), Faculty of Engineering, National University of Mar del Plata, Mar del Plata, Argentina; ^2^National Scientific and Technical Research Council (CONICET), Ministry of Science, Technology and Innovation, Buenos Aires, Argentina; ^3^Institute of Biological Research (IIB), CONICET, National University of Mar del Plata, Mar del Plata, Argentina

**Keywords:** textile wastewater, biological treatment, simulated effluents, real effluents, toxicity

## Abstract

Wastewater discharge is a matter of concern as it is the primary source of water pollution. Consequently, wastewater treatment plays a key role in reducing the negative impact that wastewater discharge produce into the environment. Particularly, the effluents produced by textile industry are composed of high concentration of hazardous compounds such as dyes, as well as having high levels of chemical and biological oxygen demand, suspended solids, variable pH, and high concentration of salt. Main efforts have been focused on the development of methods consuming less water or reusing it, and also on the development of dyes with a better fixation capacity. However, the problem of how to treat these harmful effluents is still pending. Different treatment technologies have been developed, such as coagulation-flocculation, adsorption, membrane filtration, reverse osmosis, advanced oxidation, and biological processes (activated sludge, anaerobic-aerobic treatment, and membrane bioreactor). Concerning to biological treatments, even though they are considered as the most environmentally friendly and economic methods, their industrial application is still uncertain. On the one hand, this is due to the costs of treatment plants installation and, on the other, to the fact that most of the studies are carried out with simulated or diluted effluents that do not represent what really happens in the industries. Integrated treatment technologies by combining the efficiency two or more methodologies used to be more efficient for the decontamination of textile wastewater, than treatments used separately. The elimination of hazardous compounds had been reported using combination of physical, chemical, and biological processes. On this way, as degradation products can sometimes be even more toxic than the parent compounds, effluent toxicity assessment is an essential feature in the development of these alternatives. This article provides a critical view on the state of art of biological treatment, the degree of advancement and the prospects for their application, also discussing the concept of integrated treatment and the importance of including toxicity assays to reach an integral approach to wastewater treatment.

## Introduction

There is an increasing awareness of how our actions impact the environment and that is why in the last decades a great number of environmentally friendly strategies and technologies have been developed. In addition, and as a consequence of social demand, environmental protection policies have been turning more rigorous. Industrial activity is not exempt and it is changing toward sustainable production models. Faced with this new paradigm, textile industry represents a hot spot since it is not only responsible for generating large volumes of effluents, but being also highly toxic. Among the physicochemical characteristics, the composition of the effluents is highly variable; being the concentration of the parameters very different between the studies available in literature. This type of effluent are characterized for having a wide range of pH between values of 5 and 12 (Singh and Singh, 2017; Yaseen and Scholz, 2019; Paździor and Bilińska, 2020; Samuchiwal et al., 2021), elevated chemical oxygen demand (COD) from 10 to 2250 mgO_2_ L^–1^ (Ghaly et al., 2014; Singh and Singh, 2017; Paździor and Bilińska, 2020; Samuchiwal et al., 2021), biochemical oxygen demand (BOD) among 100 and 3000 mgO_2_ L^–1^ (Işik and Sponza, 2004; Paździor and Bilińska, 2020), suspended solids, heavy metals and salts (Saratale et al., 2011; Singh and Singh, 2017; Vikrant et al., 2018; Yaseen and Scholz, 2019). Regarding to dye concentration, they are highly colored. For example, concentration of dyes varies from 10 to 250 mg L^–1^ (Ghaly et al., 2014), however, concentrations up to 600–800 mg L^–1^ have been found, and the same variability is usually found for COD and BOD (Yaseen and Scholz, 2019). This variability is a matter of concern, since investigations for the development of treatment technologies are mostly carried out with simulated effluents. Therefore, this represents an obstacle when the treatments developed at laboratory scale with simulated effluents must be applied since there is no specific composition of textile wastewater.

Otherwise, textile production must overcome a series of challenges including the development of dyes with better fixation capacity, reduction of water use, reuse of water, reduction of costs, improvement of the finish of garments and treatment processes, all of them tending to achieve a more sustainable process. Consequently, the approach to the problem must be multifactorial if we are looking to solve the impact that textile discharges cause on the environment. Not only the methodologies and models used in research must be adapted, but there must be a decision on the industrial sector, accompanied by the creation of public policies, that promote the development of these technologies.

Reasons for the change are more than enough since the negative effects in the environment are widely known. The degree of damage depends on the volume, composition and duration of the discharged wastewater. This can modify the penetration of sunlight into water bodies and change solubility of atmospheric gases, altering photosynthesis and therefore the entire aquatic ecosystem (O’Neill et al., 1999; Saratale et al., 2011; Gonçalves et al., 2017; Rather et al., 2018). In addition, the toxic, mutagenic and carcinogenic effect of several compounds present in the effluents, such as dyes, have been demonstrated (Saratale et al., 2011; Dhaouefi et al., 2018). Moreover, withdrawal of heavy metals by some chemicals used as mordents, eventually increases wastewater toxicity (Dhaouefi et al., 2018).

Since many decades ago physicochemical and biological technologies are being developed for textile wastewater treatment. Physicochemical ones have the disadvantage of being expensive; they have high chemical and energy requirements, and produce secondary byproducts, which can endanger their full-scale implementation (Dhaouefi et al., 2018). In some cases, these methods such as adsorption, chemical precipitation or electrochemical precipitation are not able to remove compounds like dyes or their metabolites from the wastewater, only transferring the pollutant from one phase to another, (Hayat et al., 2015; Singh and Singh, 2017), thus failing to solve the problem. Otherwise, biological methods are economic, simple, and environmentally friendly alternatives. Although significant toxicity reduction is observed with these treatments, pollutants mineralization is rarely achieved. As well, microorganism’s growth and catalytic activity is often inhibited by the presence of toxic substances, making difficult the extrapolation to-large-scale processes. This leads to the need of modify the natural effluent conditions to carry out research, as for example the use of simulated or diluted effluents, the addition of nutritional supplements and/or previous acclimatization. However, despite the related problems, the treatment of textile wastewater is required not only to do no harm the environment, but also to recover the water from the wastewater and recycle it for irrigation or reuse indoors the factory (Yaseen and Scholz, 2019).

## Regarding Biological Treatments

When we talk about biological treatment, we refer to strategies that use the abilities of microorganisms, plants or enzymes to remove (either by degradation or sorption) a contaminant from a matrix such as soil, sediment, air, or water (Singh and Singh, 2017), and therefore minimize the negative impact in the environment by reducing its toxicity. Among them, treatments that use microorganisms (bacteria, algae, and fungi) are the most widespread.

There is a wide range of references in which microorganisms in pure cultures are used; an *Aeromonas hydrophila* was able to reduce 72% of dyes presents in a simulated wastewater (Thanavel et al., 2019). In other work, a *Trichoderma tomentosum* reached a decolorization of 94.9% in real diluted wastewater and simulated effluent (He et al., 2018). However, nutritional supplements (such as the addition of some carbon or nitrogen sources) and/or previous acclimatization are frequently required in this kind of methodologies to improve results. Even more, achieving the sterile conditions necessary to maintain pure cultures would not be feasible due to the high costs that this would imply (Paździor et al., 2019).

In recent years, the use of mixed microbial cultures (combining several bacterial and/or fungal strains), consortia or microbial communities has gained relevance. Microbial communities have the ability to utilize many different carbon sources by expressing a range of metabolic capabilities (Eiler, 2006; Shade et al., 2012). As a result of the species diversity and the cooperation between them, the effluent molecules can be attacked at several sites by different strains and the decomposition products generated by the metabolic activity of one strain, may be used as a substrate by the other one (Eiler, 2006; Dafale et al., 2008; Holkar et al., 2016). This gives them a central role in a wide variety of biotechnological applications, like textile wastewater treatment. In contrast, pure cultures are associated to specific compounds, being less versatile in terms of their biodegradation ability (Jadhav et al., 2010; Paździor et al., 2019).

Despite the great advances in the efficiency of the developed biological treatments, there are still some limitations, since most of the researches (80%) are carried out with simulated or synthetic effluents and by the addition of nutritional sources (Table 1). For example, microbial community found by Chen et al. (2019) could reach a decolorization efficiency between 71.3 and 96.3% in a synthetic effluent (600 mg L^–1^ dye concentration), but just after the addition of 3.00 g L^–1^ of beef extract, 5.00 g L^–1^ of glucose, and 2.00 g L^–1^ peptone. Other works used even 10 g L^–1^ of glucose (Waghmode et al., 2019). Although good results are achieved in terms of reducing the chemical oxygen demand (COD) and decolorization, usually these values are for dye concentration that does not exceed the 200 mg L^–1^, and as was shown earlier dye concentration in real textile wastewater can reach until 800 mg L^–1^. In addition, it should be noted that reactor volumes in many cases are small (Valli Nachiyar et al., 2016; Abd El-Rahim et al., 2017; Hossen et al., 2019; Zahran et al., 2019). As an example, Hossen et al. (2019) studied the decolorization efficiency of different strains in simulated effluents, some of which reached efficiencies of even 90%. Since these results are promising, they were achieved using a batch reactor of 50 mL, a volume that is so far from the real one. This deviation from real situation of textile wastewater makes its subsequent application difficult. Although it is true that keeping the composition of the untreated synthetic wastewater constant is necessary to make valid comparisons of treatment system evaluations (O’Neill et al., 1999), after several decades and thousands of studies that validate the efficiency of biological treatments, it is necessary to leave behind the experimental comfort of simulated effluents to advance one more step in the development of strategies based in real effluents to begin their application.

**TABLE 1:** Comparison among different biological treatments of textile wastewater.

Effluent type	Dye concentration (mg L^–1^)	Organism	Biological treatment efficiency	References
Synthetic	32.5	Bacterial consortium	90–73.1% COD removal	Oliveira et al., 2020
Synthetic	200	Microbial consortium	31–70% COD removal	Waghmode et al., 2019
Synthetic	600	Bacterial consortium	71.3–96.3% decolorization	Chen et al., 2019
Synthetic	100	*Aeromonas hydrophila*	72% decolorization	Thanavel et al., 2019
Synthetic	200	*Alcaligenes faecalis, B. cereus, Bacillus* ssp.	90% decolorization	Hossen et al., 2019
Real	200	Microbial consortium	77.6% decolorization	Ceretta et al., 2018
Real	n.d.	Activated sludge	10–72% decolorization	Paździor and Bilińska, 2020
Real	n.d.	Bacterial consortium	70–73% decolorization	Samuchiwal et al., 2021
Synthetic and diluted real	100	*Trichoderma tomentosum*	94.9% decolorization	He et al., 2018
Synthetic	100	Bacterial consortium	97% decolorization	Chen et al., 2018
Synthetic and real	100	Microbial consortium	78% COD removal	Kurade et al., 2017

There are a few cases where real textile wastewater is used (Table 1). Samuchiwal et al. (2021) used a microbial consortium to degrade real wastewater, but a significant decreases of color of 70–73% was only achieved by the addition of yeast extract as external input and Pre-Treatment Range (PTR) effluent (with starch) as a carbon source. In the case reported by Ceretta et al. (2018), undiluted real textile wastewater was used, without the addition of an extra nutritional source. The percentage of decolorization achieved was approximately 77% of an effluent with 200 mg L^–1^ dye concentration (Ceretta et al., 2018). Nonetheless, the volume of the treated effluent is still small (120 mL) and there are no larger-scale studies about it.

Paździor and Bilińska (2020) analyzed an example of industrial textile wastewater treatment plant large-scale for the treatment of real undiluted textile wastewater. Despite the overall COD removal efficiency exceeded 93% and varied in a narrow range, the authors concluded that wastewater is only partially biodegradable. Also, the treatment plant consists in a 10 step system which raises installation costs (Paździor and Bilińska, 2020). Therefore, analyzing these reports, first we can conclude that the combination of more than one treatment methodology it would be necessary to achieve a better performance, by combining the degradation efficiency of more than one method. Second, that all the reviewed works have strengths but also weaknesses, either due to the scale in which they are carried out, the need for external inputs, pre-treatments or the use of simulated or diluted effluents. Clearly, textile effluents represent a challenge that has not yet been overcome.

## Importance of Toxicity Assessment

The removal efficiency of the pollutants in a wastewater depends on the treatment technology, the retention time and on the effluent composition. However, the decrease of the parameters could not be directly related to toxicity reduction as the byproducts could be more toxic than the parental ones. For these reasons, it is important to measure toxicity reduction of the treated wastewater by direct toxicity assays. These can warn the adverse effects of wastewater containing a mixture of pollutants. Nevertheless, a key issue represents which assay will be used. There are many classes of toxicity assays depending on the way by how the effluent will be discharged to the environment and use for example bacteria, microalgae, invertebrates, plants and fishes as sensor organisms. Many studies not only analyze organism growth rate but also mutagenic capacity of pollutants by genotoxicity tests performed on different cell lines. Also, it is important to note that most of the studies used synthetic or simulated wastewater, which may not be reflecting what could happen in a real situation with a real wastewater.

Table 2 shows examples of different toxicity assays performed. Phytotoxicity is a very fast an economic method to test toxicity. It measures seed germination, root elongation, seedling development as main parameters and is the most widespread assay (Valli Nachiyar et al., 2016; Ceretta et al., 2018; Chen et al., 2018; He et al., 2018; Waghmode et al., 2019). For other toxicity tests, as genotoxicity and zootoxicity, there are fewer studies available in literature (Paździor et al., 2017; Dhaouefi et al., 2018; Przystaś et al., 2018; Oliveira et al., 2020). The comparison of the results of phytotoxicity assay with other toxicity test, such as zootoxicity for the same treated wastewater showed differences. For example, Oliveira et al. (2020) showed no reduction of the phytotoxicity, while for zootoxicity a maximum reduction of 47% was obtained. The same was observed for the study of Przystaś et al. (2018), but with opposite results. In addition to these observations, phytotoxicity analysis of wastewater treated only by biological methods, most of the times showed about 50% or less in toxicity reduction, while when biological treatments are combined with other treatments, the toxicity reduction increase (Dhaouefi et al., 2018; Waghmode et al., 2019).

**TABLE 2:** Comparison of toxicity analysis in different researches carried out with biological treatment in textile wastewater.

Toxicity assay	Effluent type	Treatment	Toxicity reduction	References
Zootoxicity test (*Daphnia magna*) phytotoxicity test (*Lactuca sativa*)	Synthetic	Bacterial consortium	10–47% (*Daphnia magna*) 0% (*Lactuca sativa*)	Oliveira et al., 2020
Phytotoxicity *Sorghum vulgare* and *Phaseolus mungo*	Synthetic	Sequential photocatalytic and biological treatment	100% (*Phaseolus mungo*) and 90% (*Sorghum vulgare*)	Waghmode et al., 2019
Phytotoxicity *Glycine max* and *Adenanthera microsperma*	Synthetic	*Trichoderma tomentosum*	Not significant	He et al., 2018
Genotoxicity test and Phytotoxicity test (*Raphanus sativus*)	Synthetic	Anoxic-aerobic photobioreactor	Non-genotoxic and enhanced *R. sativus* seedlings	Dhaouefi et al., 2018
Phytotoxicity (*Lactuca sativa*)	Real	Bacterial consortium	50%	Ceretta et al., 2018
Zootoxicity (*Daphnia magna*) and phytotoxicity (*Lemna sp.*)	Synthetic	Pure cultures of fungi *(P. ostreatus, Gloeophyllum odoratum* and *Polyporus picipes)*	Significant reduction for phytotoxicity and a slight decrease for zootoxicity. Variable result depending on the fungus and the support used.	Przystaś et al., 2018
Phytotoxicity Black beans and Rice	Synthetic	Bacterial consortium	50–56%	Chen et al., 2018
Microtox toxicity test (*Vibrio fischeri*)	Real	Microbial consortium	96–98%	Paździor et al., 2017
Phytotoxicity (*Vigna radiata* and *Triticum aestivum*)	Synthetic and real diluted	Bacterial consortium	30% (*Vigna radiata*) and 45% (*Triticum aestivum*)	Valli Nachiyar et al., 2016

Yu et al. (2019) studied the correlation between the toxicity reduction of 12 wastewater treatment plants, from an industrial park, with the treatment process. They used a group of toxicity assays involving microorganism, phytoplankton, zooplankton, plant, and human cell lines. All the influents of wastewater treatment plants induced high toxicities. Seven from all the studied wastewater treatment plants showed a significant toxicity reduction after the treatment. However, the effluents of five of them induced higher toxicity in one or more toxic endpoints compared to the influents. Also, among all toxic endpoints evaluated low correlation coefficients were obtained, indicating that set of toxicity assays was necessary to completely characterize the toxicity and risk of wastewater in industrial parks.

Finally, a more careful analysis has to be done about the toxicity of the wastewater and that it is not enough to choose just a toxicity assay. It is important to select the test taking into account the final objectives of the treatment. For example, if the aim is to reuse the water for irrigation purpose, it will be convenient to use phytotoxicity assays, if the aspiration is the final discharge into water bodies, it may be more appropriate zootoxicity test with aquatic organisms.

## An Interdisciplinary Approach for a Genuine Textile Wastewater Treatment

Currently, focus is not on technologies that degrade and decrease color, rather on those that can produce reusable water, recuperate salt and/or dyes, fully mineralize the target contaminant and mainly reduce toxicity (Holkar et al., 2016). As we have seen so far, there are a huge diversity treatment types with their strengths and weaknesses, and when they are used separately the mentioned aims can rarely be achieved. Last years, researchers have been working in the development of sustainable water treatment strategies that efficiently combined physical, chemical and biological processes for the treatment of different wastewaters. The design of this integrated treatment processes is also gaining importance in the field of textile wastewater (Waghmode et al., 2019; Bhanot et al., 2020; Paździor and Bilińska, 2020). While in the last decades the number of investigations carried out on the subject has remained constant, in the last 5 years literature in which two or more treatment techniques are combined have quadrupled.

The effects of different combinations like physicochemical pre-treatment followed by a biological treatment or vice versa had been largely studied. For example, sequential photocatalytic and biological treatment with an artificial microbial consortium reach 100% degradation rate in a simulated textile wastewater within 4 h, also decreasing COD and phytotoxicity (Waghmode et al., 2019). In other study, a bacterial consortium isolated from a dyeing factory showed an efficacy of 77.6 ± 3.0% in decolorizing wastewater (Ceretta et al., 2018). When it was coupled with photocatalysis using ZnO/Polypyrrole during only 60 min, the total decolorization efficiency increased to 95.7% and 99.8% TOC degradation (Ceretta et al., 2020). Besides the good efficiency reached, the scaling-up of these processes is still pending. Paździor et al. (2017), investigate different combinations of chemical and biological methods, resulting the best one this combining biodegradation followed by the ozonation with an activated sludge, in a diluted real wastewater, producing the highest toxicity reduction (98%). Similar results were obtained by Aravind et al. (2016), who combined biodegradation followed by photo-assisted electro-oxidation and reaches a decolorization of 98%.

As we saw in this quick analysis, there are many technology designs that combine biological and physicochemical treatments. Researches carried out have shown that integrated treatments significantly improve the removal of organic matter, achieve higher decolorization percentages, reduce toxicity and even reach mineralization of contaminants (Oller et al., 2011; Hayat et al., 2015; Aravind et al., 2016; Paździor et al., 2017, 2019). The efficiency of studies that apply biological pre-treatments followed by physicochemical treatments has been compared with those that did it by the other way. Although there is no consensus in which is the best one, due to the characteristics of the textile effluents, greater difficulties have been observed in the effluent treatment when the physicochemical pre-treatments are employed (Paździor et al., 2019). Furthermore, in this way pre-treatment times are much longer, increasing costs due to the energy and chemical requirements (Qian et al., 2013; Aravind et al., 2016), added to the fact that degradation products can be toxic. Using biodegradation as pre-treatment has the advantage not only of being cheaper, but also due to the enzymatic microbial machine that has the versatility to accommodate any xenobiotic substances which may be present and degrade it (Saratale et al., 2011; Singh and Singh, 2017). Thus, the biological treatment contribute to the overall organic and color removal, and the physicochemical ones cooperate in completing the degradation of pollutants in a shorter time and also contributing to the sterilization of the biotreated wastewater for its subsequent discharge (Paździor et al., 2019).

The case of study of Paździor and Bilińska (2020) is important since analyzed a large-scale industrial textile wastewater treatment plant. In this plant biological, chemical and physical methods (such as filtration, ozonation, biological treatment with activated sludge) are combined. While improving of BOD_5_/COD ratio and COD reductions, the authors concluded that after a ten steps system wastewater is only partly biodegradable (Paździor and Bilińska, 2020). These examples bring out the complexity of textile wastewater.

## Conclusion

The first recorded review on the treatment of textile wastewater, which compiles different methodologies and their efficiency in the removal of dyes, dates from 1971 (Porter, 1971). But after five decades of research and with thousands of accumulated works, according to the United Nations 80% of industrial wastewaters are still being discharged into the environment without treatment (United Nations World Water Assessment Programme, 2017).

To date, some certainties have been reached but there are still a few challenges to overcome. The first ones confirm that progress has been made in the field of textile wastewater treatment:

(i) the use of consortia or microbial communities makes the biodegradation of complex compounds possible;
(ii) the use of combined toxicity assays is necessary to determine the efficiency of the applied treatments;
(iii) the development of integrated treatments by combining two or more methodologies allows to achieve higher percentages of degradation.

Among the challenges that are pending to be solved: the study of treatment methodologies using real effluents, the scaling up of the processes with the corresponding cost analysis, the development of economically attractive alternatives to achieve adoption by the industrial sector, and finally reducing water consumption by reusing it.

It is evident that the combination of physical, chemical and biological methods must be used to carry out a successful treatment, not only in terms of reducing dye concentration and COD, but also for reducing pH, BOD, toxicity. All this objectives will be reached only if the work is carried out in an interdisciplinary way, where scientific evidence, engineering dimensions, but also economic and political ones are taken into account. The implementation of this type of alternatives will only be possible with the support of public policies that value and accompany by supporting, the implementation of effluent treatment strategies for the environment protection.

## Author Contributions

All authors listed have made a substantial, direct and intellectual contribution to the work, and approved it for publication.

## Conflict of Interest

The authors declare that the research was conducted in the absence of any commercial or financial relationships that could be construed as a potential conflict of interest.
